# Fat‐Corrected Non‐Gaussian Diffusion MRI for Liver Fibrosis Assessment in Metabolic Dysfunction‐Associated Steatotic Liver Disease

**DOI:** 10.1002/jmri.70148

**Published:** 2025-10-24

**Authors:** Omaïma Saïd, Sabrina Doblas, Valérie Paradis, Pierre Bedossa, Dominique Valla, Cédric Laouénan, Laurent Castera, Bernard E. Van Beers, Philippe Garteiser

**Affiliations:** ^1^ Center for Research on Inflammation Inserm U1149 Université Paris Cité Paris France; ^2^ Histopathology Department Beaujon University Hospital, Assistance Publique—Hôpitaux de Paris Clichy France; ^3^ Hepatology Department Beaujon University Hospital, Assistance Publique—Hôpitaux de Paris Clichy France; ^4^ Infection, Antimicrobials, Modelling, Evolution (IAME), Inserm U1137 Université Paris Cité ‐ Université Sorbonne Paris Nord Paris France; ^5^ Département d'Epidémiologie Biostatistique et Recherche Clinique Bichat University Hospital, Assistance Publique—Hôpitaux de Paris Paris France; ^6^ Radiology Department Beaujon University Hospital, Assistance Publique—Hôpitaux de Paris Clichy France

**Keywords:** diffusion‐weighted MRI, intravoxel fat correction, liver fibrosis, MASLD, MR elastography, non‐gaussian diffusion

## Abstract

**Background:**

In patients with metabolic dysfunction‐associated steatotic liver disease (MASLD), non‐Gaussian diffusion—weighted imaging (DWI) has been proposed for the diagnosis of liver fibrosis, but its measurement is partially confounded by steatosis. We therefore asked whether a fat‐corrected approach could improve fibrosis assessment.

**Purpose:**

To evaluate the diagnostic performance of non‐Gaussian diffusion coefficients for the assessment of fibrosis in MASLD patients with a method accounting for intravoxel fat.

**Study Type:**

Prospective single‐center cross‐sectional study.

**Population:**

A total of 289 participants with Type 2 diabetes, hepatic steatosis, and elevated aminotransferases were enrolled from October 2018 to June 2021. Among them, 222 participants (mean age 59 ± 10 years; 149 men) underwent liver biopsy and MRI and were included in the final analysis.

**Field Strength/Sequence:**

3 T, DWI using spin‐echo echo‐planar imaging, MR elastography (MRE) using gradient echo sequence and fat fraction imaging using a multiple gradient echoes sequence.

**Assessment:**

Diffusion coefficients were estimated using two non‐Gaussian models: a shifted apparent diffusion coefficient (sADC) and a non‐linear least squares fit (ngADC), both computed without and with intravoxel fat correction (corr) using fat fraction on PDFF. Fibrosis was staged histologically. Quantitative parameters were compared across fibrosis stages. Diagnostic performance for F0 versus ≥ F1 was evaluated and compared to liver stiffness on MRE.

**Statistical Tests:**

Group comparisons used Kruskal‐Wallis tests (*α* = 0.05), and diagnostic performance was assessed via receiver operating characteristic (ROC) curve analysis with 95% confidence intervals, with *p* < 0.05 considered statistically significant.

**Results:**

ngADC_corr_ was significantly different between fibrosis stages (Kruskal–Wallis *p* < 0.05). ROC curve analysis indicated comparable performance in discriminating fibrosis stages F0 versus F1–F4 for ngADC_corr_ and stiffness (AUC = 0.66, 95% CI: [0.59, 0.7], *p* < 0.05 and 0.68 [0.62, 0.74], *p* < 0.05, respectively).

**Data Conclusion:**

Fit‐based non‐Gaussian DWI with fat correction could potentially be used with similar diagnostic accuracy as MRE for detecting fibrosis in patients with MASLD.

**Evidence Level:**

3.

**Technical Efficacy:**

Stage 2.

## Plain Language Summary

Liver disease related to fat accumulation is common in people with metabolic problems, such as Type 2 diabetes. Detecting early liver scarring (fibrosis) is important, but standard MRI measurements can be affected by fat in the liver. In this study, researchers tested a new MRI method that adjusts for liver fat when measuring water movement in the liver. They found that this fat‐corrected approach could identify liver fibrosis similarly to a widely used but more complex technique called MR elastography. These findings suggest that fat‐corrected MRI may help doctors detect liver fibrosis more reliably in patients with fatty liver disease.

## 1 Introduction

In metabolic dysfunction‐associated steatotic liver disease (MASLD), fibrosis represents an important predictor of long‐term survival [1]. Diffusion‐weighted imaging (DWI) provides a measure of molecular diffusion of water in tissues, and changes in tissue microstructure can lead to variations in the apparent diffusion coefficient (ADC) [2]. The measurement of these variations may be helpful for staging liver fibrosis [3, 4, 5, 6, 7, 8]. However, compared with MR elastography (MRE) and despite being technically simpler to acquire, conventional DWI has shown only moderate accuracy to stage liver fibrosis [4, 9].

Improving accuracy may depend on the use of more advanced DWI methods, such as diffusion kurtosis imaging (DKI) [2]. In conventional DWI, water molecule diffusion is assumed to follow a Gaussian distribution even in the presence of barriers, leading to an “apparent” diffusion coefficient. Conversely, DKI explicitly introduces a second order term in ${\left(b\cdot \mathrm{ADC}\right)}^{2}$ along with a scaling coefficient k to account for the non‐gaussian nature of diffusion that arises in the presence of barriers. As predicted in the kurtosis model, these deviations only become apparent at high values of *b* (typically > 1000 s/mm^2^), but they could inform on the microstructural complexity of tissues [10, 11]. Despite requiring high *b*‐values, non‐Gaussian diffusion imaging can still be readily achieved on clinically accessible MRI scanners [2, 12], and has the advantage of not requiring any external hardware. To further ease the applicability of non‐Gaussian diffusion imaging in the clinic, a simplified method involving the measurement of a “shifted ADC” (sADC) has been recently proposed [13, 14]. This method, relying on an index value of signal intensity at two key *b*‐values of 200 and 1500 s/mm^2^, has been validated for liver fibrosis staging using MRE as reference examination [13, 14]. However, the accuracy of non‐Gaussian diffusion‐based imaging and sADC methods for liver fibrosis staging has not been compared.

Another possible reason for the moderate accuracy of conventional DWI for fibrosis staging is the influence of fat on the apparent diffusion coefficient. Liver steatosis confounds the estimation of water diffusion coefficients and interferes with fibrosis staging [4, 15, 16, 17]. Because of this dependency of diffusion measurements on liver steatosis, sADC and a proposed fat‐corrected version of sADC have been shown to have no dependence on fibrosis in a population of patients with MASLD [18]. An alternate fat correction scheme has been recently proposed in a letter to the editor, but this method has not yet been tested in clinical studies [19].

The aim of this study was to evaluate the diagnostic performance of non‐Gaussian diffusion for liver fibrosis staging by comparing different approaches, including a fit‐based and fat‐corrected method, in patients with biopsy‐proven MASLD.

## 2 Materials and Methods

### 2.1 Participants

The full inclusion and exclusion criteria have been detailed elsewhere [20]. Briefly, the eligible population consisted of adult patients with Type 2 diabetes mellitus and a suspicion of MASLD warranting a biopsy. Patients with other causes of liver disease such as excessive alcohol intake, viral hepatitis, or hemochromatosis were excluded. Participants with incomplete MRE/DWI/PDFF examinations (due to technical or recruitment issues), with regions of interest smaller than 2.0 cm^2^, or without available histopathological results were excluded from the analysis. The participants were consecutively recruited within the “QuidNASH” clinical trial (ClinicalTrials.gov NCT03634098) from four hospitals. The study was nested within this trial and was limited to patients from a single MRI center (Beaujon university hospital). MRI measurements defined below were used as index tests, and histological analyses as detailed below were used as reference tests.

### 2.2 MRI Acquisition

The MR images were obtained on a 3 T Ingenia system using a multi‐element abdominal receive coil (Philips, Eindhoven, The Netherlands). Participants fasted for at least 8 h before the examination. The examination included, besides a DWI sequence, a MRE acquisition for determining liver stiffness and a multiecho gradient‐echo sequence for determining the proton density fat fraction (PDFF). Other quantitative or anatomical sequences were also performed in these patients as part of the trial, including T1 and T2‐weighted sequences.

The diffusion dataset was obtained in free breathing with a spin‐echo single shot echo‐planar imaging sequence and SENSE parallel imaging with an acceleration factor of three. Field of view was 32 × 38 cm, with acquisition voxel size of 3.25 × 3.25 mm (reconstructed to 0.89 × 0.89 mm). Twelve slices were acquired (8 mm thickness). Echo time was 70 ms (31.4 Hz bandwidth and 2690 Hz in the echo‐planar direction), and repetition time was 2.5 s, for a total acquisition time of 2 min 30 s. Spectral‐attenuated inversion recovery (SPAIR) fat suppression was applied. Diffusion encoding was performed with six *b*‐values (0, 50, 100, 200, 800, and 1500 s/mm^2^ averaged 1, 1, 1, 1, 4, and 6 times, respectively), using trapezoidal gradient pulses of 12.2 ms duration separated by 35.2 ms composited from the three physical gradient orientations into a single diagonal gradient encoding direction. In each patient, a region of interest was positioned on a central slice of the right liver lobe, overlapping the MRE slice stack by selecting the largest area available while avoiding large vessels and organ edges. Region of interest (ROI) positioning was performed by a physicist (PG) with 15 years of experience in abdominal imaging under supervision from a radiologist (BVB), blind to MRE and histological results. Inter‐observer reproducibility was evaluated in a subset of 20 patients. A second observer (OS) independently re‐drew the ROIs, blinded to the results of the first observer.

MRE was acquired using a gradient echo sequence at a mechanical frequency of 60 Hz with four mechanical phases. Mechanical actuation was performed with a commercially available device (Resoundant). Three 10 mm thick slices were acquired, at a final resolution of 1.2 mm. Motion was encoded in the through‐slice direction. Acquisition duration was three breath holds of 15 s each. Reconstruction of stiffness maps (kPa) was performed with the algorithm provided by the manufacturer [21]. ROIs were positioned by a physicist (PG) with 15 years of experience in abdominal imaging under the supervision of a radiologist (BVB) on all available slices, by selecting the largest area available while avoiding low‐confidence regions marked as gridded regions on the manufacturer‐generated images, large vessels, and organ edges. The stiffness value for each patient was obtained by averaging the stiffness values of all selected voxels.

PDFF was calculated with a multi‐echo Dixon sequence (mDixonQuant, Philips Healthcare). Six echoes were obtained at 1.7 mm in‐plane resolution, 4 mm slice thickness, and a 3° flip angle. PDFF values were extracted from the manufacturer‐generated PDFF maps by averaging the values of three circular regions of interest positioned in different segments of the liver on the same slices as in the diffusion images [22, 23, 24].

### 2.3 DWI Processing

To evaluate liver diffusivity, five different ADCs were computed for each participant using fit‐based and index‐based non‐Gaussian models, with and without fat correction. All calculations were performed using “DiffuMapper”, a custom‐built application using the lmmin C++ library controlled via a MATLAB‐based graphical interface. The calculated metrics were the Gaussian diffusion coefficient obtained from a mono‐exponential model, ADC, the non‐Gaussian ADC from a non‐linear least squares fit (no fat correction), ngADC, the index‐based ADC from Le Bihan's method (no fat correction), sADC, the non‐Gaussian ADC from a non‐linear least squares fit with a fat correction term (Hansmann model), ${\text{ngADC}}_{\text{corr}}$, the index‐based ADC with fat correction (Hanniman's method), ${\text{sADC}}_{\text{corr}}^{\text{Hanniman}}$, and the index‐based ADC with fat correction (Le Bihan's method), ${\text{sADC}}_{\text{corr}}^{\mathrm{Le}\;\text{Bihan}}$ [14, 16, 18]. More specifically, mono‐exponential ADC and ngADC were calculated by non‐linear least‐squares fitting of signal intensities at all b‐values with the Levenberg–Marquardt algorithm, using the following models:

(1) $$S\left(b\right)=S_{0}\cdot e^{-b\cdot \mathrm{ADC}}$$

(2) $$S\left(b\right)=S_{0}\cdot e^{-b\cdot \text{ngADC}+\frac{1}{6}\left(b^{2}\cdot {\text{ngADC}}^{2}\cdot k\right)}$$

where S(*b*) is the signal intensity at *b*‐value *b* (s/mm^2^), *S*_0_ is the signal intensity at *b* = 0, ADC is the monoexponential Gaussian ADC (mm^2^/s), ngADC is the non‐Gaussian diffusion coefficient (mm^2^/s) and *k* is the kurtosis (dimensionless).

The sADC was calculated as follows:

(3) $$\text{sADC}=\frac{\ln\left(\frac{S_{b_{200}}}{S_{b_{1500}}}\right)}{b_{1500}-b_{200}}$$

where $S_{b_{200}}$ and $S_{b_{1500}}$ are the signals acquired at *b* values *b*_200_ = 200 s/mm^2^ and *b*_1500_ = 1500 s/mm^2^ respectively [14].

The following coefficients were calculated using fat correction. Indeed in the presence of intravoxel fat, the measured ADC does not precisely reflect the true ADC of water, because imperfections in fat suppression result in small and variable percentage of signal still originating from fat [16]. To compensate for this, additional fat correction was performed by fitting the data according to Hansmann's model that explicitly accounts for a fat compartment in which diffusion is assumed negligible. Hence the ngADCcorr was calculated as follows:

(4) $$S\left(b\right)=A\left[\left(1-\eta \right)e^{-\frac{\mathrm{TE}}{T_{2}^{w}}}e^{-b\cdot {\text{ngADC}}_{\text{corr}}+\frac{\left(b^{2}\cdot {\text{ngADC}}_{\text{corr}}^{\text{2}}\cdot k_{w}\right)}{6}}+\alpha \eta e^{-\frac{\mathrm{TE}}{T_{2}^{f}}}\right]$$

where *η* is the proton density fat fraction (as measured from mDixonQuant), *α* is the residual fat signal (8.7% [16]), $T_{2}^{w}$ and $T_{2}^{f}$ are the T2 relaxation times for water (23 ms) and fat (62 ms), and TE is the echo time (ms) [16].

The sADC corrected for fat were calculated using the fat correction scheme proposed by Hanniman ($\mathrm{sAD}C_{\text{corr}}^{\text{Hanniman}}$) and the fat correction scheme proposed by Le Bihan ($\mathrm{sAD}C_{\text{corr}}^{\mathrm{Le}\;\text{Bihan}}$), where the *b* = 0 signal is incorporated to better estimate water ADC [18, 19]. These coefficients were calculated as follows, using variables as defined previously:

(5) $$\mathrm{sAD}C_{\text{corr}}^{\text{Hanniman}}=\frac{1}{b_{1500}}\cdot \ln\left(\frac{\left(1-\eta \right)\cdot e^{-\mathrm{TE}/T_{2}^{w}}}{\frac{S_{b_{1500}}}{S_{b_{200}}}\cdot \left(\left(1-\eta \right)\cdot e^{-\mathrm{TE}/T_{2}^{w}}\cdot e^{-b_{200}\cdot \text{sADC}}+\alpha \cdot \eta \cdot e^{-\mathrm{TE}/T_{2}^{f}}\right)-\alpha \cdot \eta \cdot e^{-\mathrm{TE}/T_{2}^{f}}}\right)$$

and

(6) $$\mathrm{sAD}C_{\text{corr}}^{\text{LeBihan}}=\frac{b_{1500}\cdot \mathrm{AD}C_{w}^{0-b_{1500}}-b_{200}\cdot \mathrm{AD}C_{w}^{0-b_{200}}}{b_{1500}-b_{200}}$$

with

(6a) $${\text{ADCw}}^{0-b}\;=\frac{1}{b}\ln\left(\frac{\begin{array}{c} \left(1-\eta \right)\;e^{-\frac{\mathrm{TE}}{T_{2}^{w}}}\\ \; \end{array}}{\frac{\mathrm{Sb}}{S0}\left(\left(1-\eta \right)e^{-\frac{\mathrm{TE}}{T_{2}^{w}}}+\alpha \eta e^{-\frac{\mathrm{TE}}{T_{2}^{f}}}\right)-\alpha \eta e^{-\frac{\mathrm{TE}}{T_{2}^{f}}}}\right)$$

### 2.4 Histology

Transcapsular or transjugular liver biopsies performed according to the standard technical procedure of the clinical center were obtained on the same day as the MRI session. Transcapsular biopsies were performed in the right liver lobe, and transjugular biopsies were performed by accessing the right hepatic vein through a catheter inserted into the right jugular vein. Histological evaluations of the biopsies were conducted in a centralized, blinded fashion by a pathologist (PB), blinded to the MRI results, using the routine staining protocol of the clinical center. According to the nonalcoholic steatohepatitis clinical research network, liver steatosis was graded as S0 (absence of steatotic hepatocytes), S1 (5%–33% of hepatocytes containing lipid vesicles), S2 (34%–66% of hepatocytes containing lipid vesicles), or S3 (> 66% hepatocytes containing lipid vesicles), S2 and S3 being defined as moderate to severe steatosis [25]. Fibrosis was staged as F0 for absence of fibrosis, F1 for perisinusoidal fibrosis, F2 for perisinusoidal and portal/periportal fibrosis, F3 for septal or bridging fibrosis, and F4 for cirrhosis.

### 2.5 Statistical Analysis

Diffusion‐based variables were initially assessed for their association with steatosis grade and fibrosis stage using Kruskal–Wallis tests. Linear regression was then used to evaluate the relationship between diffusion coefficient and stiffness. Coefficients of determination *R* ^2^ and associated *p* values were calculated.

The diagnostic performance of diffusion‐based variables for fibrosis was determined using histology‐determined fibrosis stage as reference test, and compared against the diagnostic performance of stiffness. This analysis employed Kruskal–Wallis tests with post hoc Conover comparisons. Kruskal–Wallis effect size was estimated with $\eta_{KW}^{2}$, computed as (*H*−*k* + 1)/(*n*−*k*), where *H* is the Kruskal–Wallis statistic, *k* is the number of groups and n the total number of observations, and was qualified as “small”, “moderate” or “large” according to values < 0.06, < 0.4 or ≥ 0.14, respectively [26]. Receiver operating characteristic (ROC) curve analysis was also carried out. Performance was evaluated for all possible dichotomizations of fibrosis severity (*F* = 0 versus (vs) *F* > 0, *F* ≤ 1 vs. *F* > 1, *F* ≤ 2 vs. *F* > 2, *F* ≤ 3 vs. F4). The AUC was compared to that of stiffness using DeLong tests. Agreement between observers was quantified using the Intraclass Correlation Coefficient (ICC) using a two‐way random‐effects model for absolute agreement [ICC(2,1)] and report ICC values with their 95% confidence intervals. ICCs were qualified as poor, fair, good or excellent for intervals 0–0.39, 0.40–0.59, 0.60–0.74 and 0.75–1.00, respectively [27]. Throughout the statistical analysis, a *p* value threshold of 0.05 was used to determine significance. All statistical tests were performed with MedCalc (Medcalc Software).

## 3 Results

### 3.1 Participants

**FIGURE 1:**
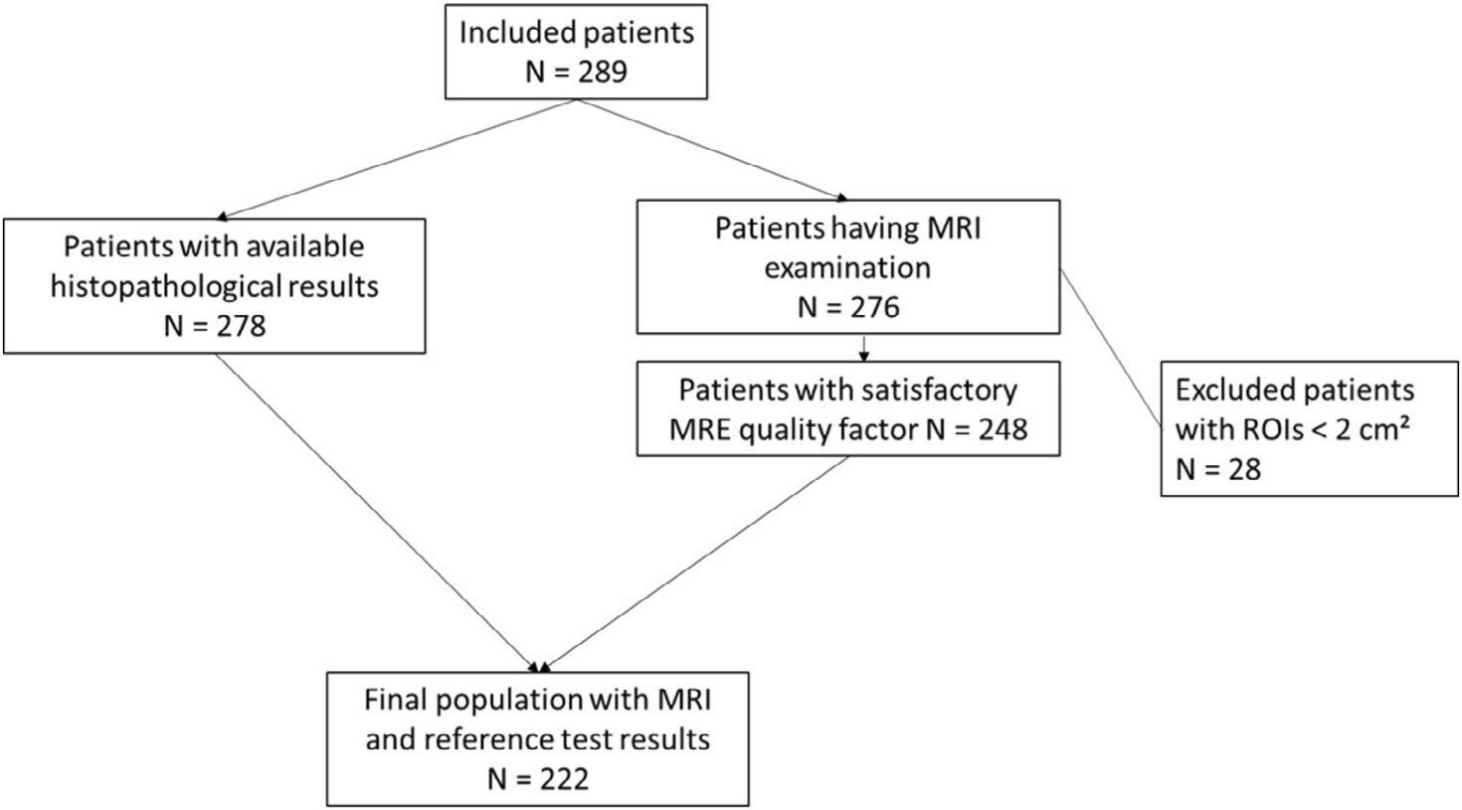
Flow chart of study participants.

Two‐hundred and eighty‐nine adult participants with Type 2 diabetes as defined by the American diabetes association [28] and MASLD were included in this institutional review board approved prospective study between October 2018 and June 2021, after obtaining written informed consent. The final study population included 222 participants (mean age 59 ± 10 years; 149 men) with complete histologic and MRI data (Figure 1, Table 1). Biopsies were transcapsular in 151 patients and transjugular in 71 patients. Fibrosis staging was F0 (*n* = 46), F1 (*n* = 61), F2 (*n* = 40), F3 (*n* = 54) and F4 (*n* = 21). Steatosis grading showed S0 (*n* = 6), S1 (*n* = 62), S2 (*n* = 119) and S3 (*n* = 35). Marked steatosis (S2 or S3) was observed in $\frac{21}{46}$ of F0 patients, $\frac{48}{61}$ of F1 patients, $\frac{33}{40}$ of F2 patients, $\frac{42}{54}$ of F3 patients and $\frac{10}{21}$ of F4 patients.

**TABLE 1:** Population characteristics.

Demographics	
Sample size	222
Mean ± standard deviation age	59 ± 10 years
Gender	67% men
Clinical	
BMI	31.5 [18 45] Kg/m^2^
Histology	
Transcapsular	151 (68%)
Transjugular	71 (32%)
Steatosis grade	6 S0, 62 S1, 119 S2, and 35 S3
Fibrosis stage	46 F0, 61 F1, 40 F2, 54 F3, and 21 F4
MRI	
Stiffness (kPa)	3.2[1.6 6.8]
F0	2.8 [1.7 4.2]
F1	2.8 [1.6 4.6]
F2	3.0 [2.0 5.0]
F3	3.7 [1.8 5.9]
F4	5.1 [1.7 6.8]
PDFF (%)	15.6 [2.9 45.1]
S0	5.8 [3.3 26.2]
S1	10.2 [3.7 24.7]
S2	17.0 [2.9 40.6]
S3	28.0 [7.2 45.1]
F0	14.7 [3.3 45.1]
F1	16.9 [2.9 43.2]
F2	17.6 [3.7 41.2]
F3	16.7 [4.2 35.1]
F4	9.7 [3.7 25.5]

*Note*: Demographic, clinical, histological, and MRI characteristics of the study population. Age and BMI are presented as mean ± standard deviation. Stiffness and PDFF are presented as median (interquartile range). Categorical variables are presented as number of patients, with percentages in parentheses.

Descriptive statistics for each of the five diffusion parameters and for MRE stiffness are provided across fibrosis stages in Table 2 and across steatosis grades in Table 3. ADC values decreased with increasing fibrosis but showed stronger variation across steatosis grades. MRE stiffness increased predominantly with fibrosis.

**TABLE 2:** Descriptive statistics (median [min–max]) of stiffness and diffusion variables (sADC, $\mathrm{sAD}C_{\text{corr}}^{\text{Hanniman}}$, $\mathrm{sAD}C_{\text{corr}}^{\mathrm{Le}\;\text{Bihan}}$, ngADC, $\text{ngAD}C_{\text{corr}}$ and mono‐exponential ADC) by fibrosis stage.

Fibrosis stage	F0	F1	F2	F3	F4
MRI parameter					
Stiffness (kPa)	2.8 [1.7–4.2]	2.8 [1.6–4.6]	3.0 [2.0–5.0]	3.7 [1.8–5.9]	5.1 [1.7–6.8]
sADC (×10^−4^ mm^2^/s)	5.41 [2.21–9.62]	5.43 [2.17–8.70]	5.03 [2.78–8.61]	5.43 [2.26–7.60]	6.17 [2.07–10.3]
${\text{sADC}}_{\text{corr}}^{\text{Hanniman}}$ (×10^−4^ mm^2^/s)	6.29 [2.84–10.1]	7.01 [2.48–10.8]	6.60 [3.38–11.1]	6.33 [2.64–11.0]	7.00 [2.60–11.5]
${\text{sADC}}_{\text{corr}}^{\mathrm{Le}\;\text{Bihan}}$ (×10^−4^ mm^2^/s)	6.95 [3.03–11.7]	7.88 [2.51–14.1]	7.02 [4.48–13.7]	6.87 [2.84–12.9]	7.67 [2.99–12.5]
ngADC (×10^−4^ mm^2^/s)	11.3 [2.87–23.8]	11.0 [3.57–21.1]	9.63 [3.60–20.7]	10.1 [4.00–23.7]	12.5 [4.14–18.7]
ngADC_corr_ (×10^−4^ mm^2^/s)	19.7 [13.5–29.5]	19.1 [15.0–23.9]	18.1 [14.9–24.9]	18.3 [14.1–27.9]	18.5 [13.9–23.5]
Mono‐exponential ADC (×10^−4^ mm^2^/s)	10.3 [2.49–21.1]	9.48 [3.59–16.9]	8.61 [3.70–19.9]	8.85 [3.72–19.2]	9.60 [3.70–19.0]

**TABLE 3:** Descriptive statistics (median [min–max]) of stiffness and diffusion variables (sADC, $\mathrm{sAD}C_{\text{corr}}^{\text{Hanniman}}$, $\mathrm{sAD}C_{\text{corr}}^{\mathrm{Le}\;\text{Bihan}}$, ngADC, $\text{ngAD}C_{\text{corr}}$ and mono‐exponential ADC) by steatosis grade.

Steatosis grade	S0	S1	S2	S3
MRI parameter				
Stiffness (kPa)	3.0 [1.9–5.2]	3.2 [1.7–6.8]	3.1 [1.6–6.2]	3.5 [2.0–5.3]
sADC (×10^−4^ mm^2^/s)	7.93 [5.71–9.62]	5.88 [2.35–10.3]	5.40 [2.18–7.84]	4.69 [2.07–7.20]
${\text{sADC}}_{\text{corr}}^{\text{Hanniman}}$ (×10^−4^ mm^2^/s)	8.50 [5.98–10.1]	6.65 [2.48–11.5]	6.59 [2.55–11.1]	6.59 [2.55–11.0]
${\text{sADC}}_{\text{corr}}^{\mathrm{Le}\;\text{Bihan}}$ (×10^−4^ mm^2^/s)	8.75 [6.11–10.3]	7.01 [2.51–12.6]	7.19 [2.84–13.7]	7.88 [2.99–14.1]
ngADC (×10^−4^ mm^2^/s)	14.8 [11.7–22.8]	11.8 [3.91–23.8]	10.7 [3.57–20.7]	8.23 [2.87–16.0]
ngADC_corr_ (×10^−4^ mm^2^/s)	19.0 [16.1–23.0]	18.8 [13.5–29.5]	18.7 [14.1–24.9]	18.4 [14.7–23.9]
Mono‐exponential ADC (×10^−4^ mm^2^/s)	11.5 [10.0–15.3]	9.86 [3.93–2.12]	8.76 [3.56–19.9]	7.94 [2.49–14.9]

For all diffusion models, comparisons between corrected and uncorrected ADC (sADC vs. ${\text{sADC}}_{\text{corr}}^{\text{Hanniman}}$, sADC vs. ${\text{sADC}}_{\text{corr}}^{\mathrm{Le}\;\text{Bihan}}$ and ngADC vs. ngADC_corr_) values showed significant differences (Mann–Whitney test, *p* < 0.05).

Steatosis, as estimated with PDFF, significantly differed among fibrosis stages (Figure S1).

### 3.2 Reproducibility

For ngADC_corr_, the ICC was 0.918 (95% CI 0.730–0.971, *n* = 20, two observers), indicating excellent reproducibility. These results demonstrate that the ROI delineation for ngADC_corr_ is highly reproducible between observers, supporting the robustness of the quantitative measurements used in this study.

### 3.3 Variables of Interest for Fibrosis Diagnosis

**FIGURE 2:**
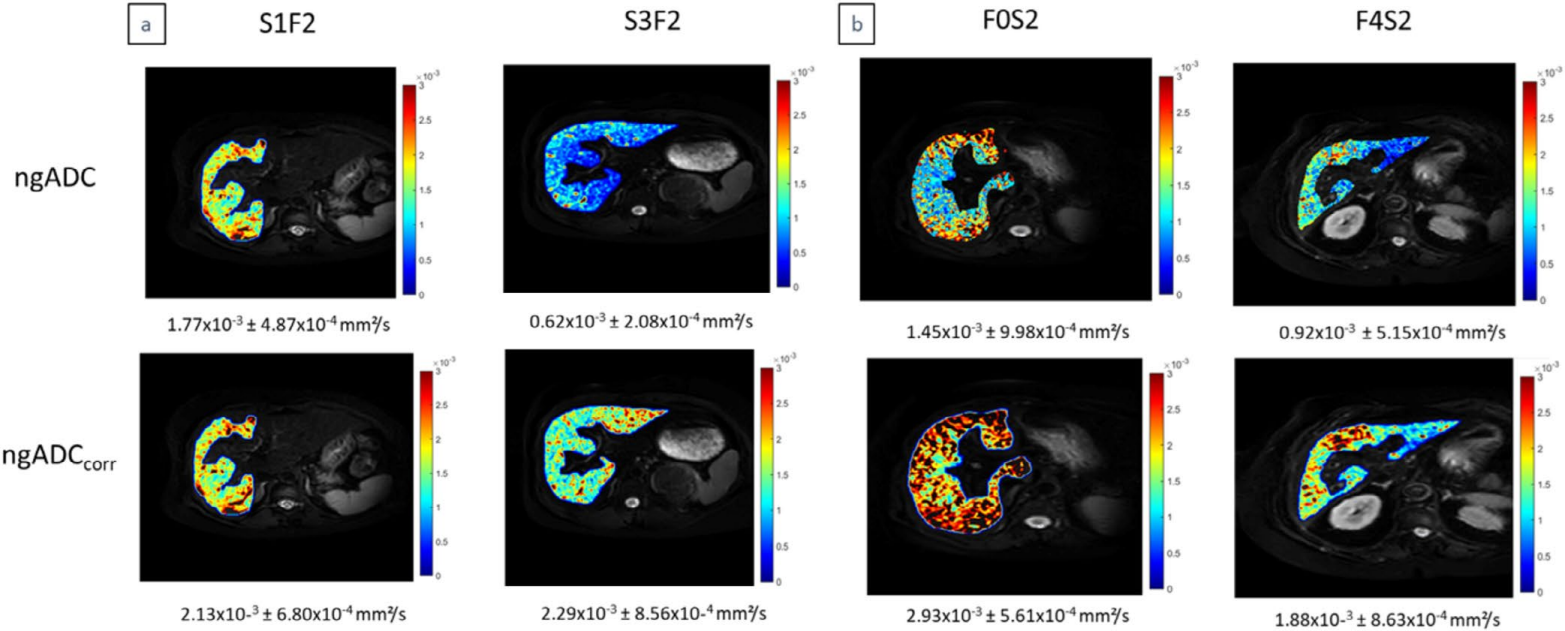
Representative parametric maps of ngADC (at the top) and ngADCcorr (at the bottom); (a): The effect of steatosis is depicted in two patients with identical F2 fibrosis stage but varying steatosis grade; (b): The effect of fibrosis is depicted in two patients with identical steatosis grade (S2) but varying fibrosis stage. In the uncorrected coefficient map, differences of ngADC are seen for steatosis (1.77 × 10^−3^ vs. 0.62 × 10^−3^ mm^2^/s), while in the corrected coefficient map, differences of ngADCcorr are seen for fibrosis (2.93 × 10^−3^ vs. 1.88 × 10^−3^ mm^2^/s).

Without fat correction, a significant difference in sADC, ngADC and mono‐exponential ADC (*p* < 0.05) was observed between steatosis grades (Table 3 and Figure 2). ADCs with fat correction did not show significant differences between steatosis grades (*p* = 0.13, *p* = 0.19 and *p* = 0.98 respectively for ${\text{sADC}}_{\text{corr}}^{\text{Hanniman}}$, ${\text{sADC}}_{\text{corr}}^{\mathrm{Le}\;\text{Bihan}}$ and ngADC_corr_). The proposed diffusion coefficient ngADC_corr_ demonstrated significant differences between fibrosis stages (*p* < 0.05, $\eta_{KW}^{2}$ = 0.049: small effect size), while none of the other parameters including fat‐corrected coefficients (${\text{sADC}}_{\text{corr}}^{\mathrm{Le}\;\text{Bihan}}$ and ${\text{sADC}}_{\text{corr}}^{\text{Hanniman}}$), were significantly different between fibrosis stages (*p* = 0.08 and *p* = 0.06 respectively). Stiffness as the reference imaging parameter displayed significant differences between fibrosis stages (*p* < 0.05, $\eta_{KW}^{2}$ = 0.267: large effect size) but not between steatosis grades (*p* = 0.84).

In the multivariate analysis, ngADC_corr_ identified fibrosis as independent variable, while uncorrected methods (sADC, ngADC and ADC mono‐exponential) had steatosis as the sole significant independent variable. Index‐based methods ${\text{sADC}}_{\text{corr}}^{\text{Hanniman}}$ and ${\text{sADC}}_{\text{corr}}^{\mathrm{Le}\;\text{Bihan}}$ did not display dependence with steatosis or fibrosis, while MRE had fibrosis as significant independent variable (*p* < 0.05).

In multivariate analysis, only ngADC_corr_ remained significantly associated with fibrosis (Table 4); therefore, subsequent analyses focused exclusively on this parameter (Table 5).

**TABLE 4:** Identification of variables with potential diagnostic value for fibrosis.

MRI parameter	Kruskal–Wallis test statistic (*p*), fibrosis stage	Kruskal‐Wallis test statistic (*p*), Steatosis grade	Multivariate linear regression, independent variable and *p*
Stiffness	61.9 (*p* < 0.05)	0.85 (*p* = 0.84)	Fibrosis (*p* < 0.05)
sADC	8.5 (*p* = 0.08)	28.6 (*p* < 0.05)	Steatosis (*p* < 0.05)
${\text{sADC}}_{\text{corr}}^{\text{Hanniman}}$	8.2 (*p* = 0.08)	5.6 (*p* = 0.13)	None
${\text{sADC}}_{\text{corr}}^{\mathrm{Le}\;\text{Bihan}}$	9.2 (*p* = 0.06)	5.7 (*p* = 0.13)	None
ngADC	7.4 (*p* = 0.11)	27.9 (*p* < 0.05)	Steatosis (*p* < 0.05)
${\text{ngADC}}_{\text{corr}}$	14.7 (*p* < 0.05)	0.21 (*p* = 0.98)	Fibrosis (*p* < 0.05)
ADC mono‐exponential	6.1 (*p* = 0.19)	25.3 (*p* < 0.05)	Steatosis (*p* < 0.05)

*Note*: Kruskal–Wallis statistic and *p* values from the Kruskal–Wallis (KW) tests. Results of post hoc analyses are not shown in this table.

Abbreviations: ngADC, non‐Gaussian ADC calculated with a fit‐based approach; ngADCcorr, non‐Gaussian ADC calculated with a fat‐corrected, fit‐based approach; sADC, index‐based apparent diffusion coefficient (ADC); ${\text{sADC}}_{\text{corr}}^{\text{Hanniman}}$, sADC calculated with Hanniman's fat correction; ${\text{sADC}}_{\text{corr}}^{\mathrm{Le}\;\text{Bihan}}$, sADC calculated with Le Bihan's fat correction.

**TABLE 5:** ROC analysis for fibrosis staging.

	*N*	Stiffness AUC [95% confidence interval] *p*	${\text{ngADC}}_{\text{corr}}$ AUC [95% confidence interval] *p*	DeLong test *p*
F0 vs. F1F2F3F4	222 (46 vs. 176)	0.68 [0.62 0.74] *p* < 0.05	0.66 [0.59 0.71] *p* < 0.05	*p* = 0.65
F0F1 vs. F2F3F4	222 (107 vs. 115)	0.75 [0.69 0.81] *p* < 0.05	0.63 [0.57 0.69] *p* < 0.05	*p* < 0.05
F0F1F2 vs. F3F4	222 (147 vs. 75)	0.80 [0.74 0.85] *p* < 0.05	0.60 [0.54 0.67] *p* < 0.05	*p* < 0.05
F0F1F2F3 vs. F4	222 (201 vs. 21)	0.85 [0.80 0.90] *p* < 0.05	0.60 [0.53 0.67] *p* = 0.11	*p* < 0.05

Abbreviation: ngADCcorr, non‐Gaussian apparent diffusion coefficient calculated with a fat‐corrected, fit‐based approach.

### 3.4 Correlation With Stiffness

**FIGURE 3:**
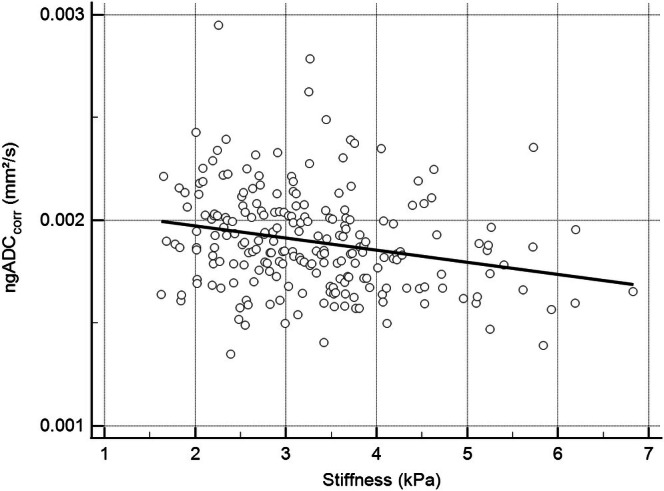
Linear regression plot of ngADC_corr_ versus stiffness (R^2^ = 0.24, *p* < 0.05). The slope (−5.9 × 10^−5^ mm^2^·s^−1^·kPa^−1^ 95% CI [−9.1 × 10^−5^, −2.7 × 10^−5^]) was significantly different from zero (*p* = 0.0004). Significant correlation was found between the two parameters.

ngADC_corr_ was negatively correlated with stiffness (*R* ^2^ = 0.24, *p* < 0.05). The slope (−6 × 10^−5^ mm^2^·s^−1^·kPa^−1^, 95% CI [−9 × 10^−5^, −3 × 10^−5^]) was significantly different from zero (Figure 3).

### 3.5 Diagnostic Performance for Liver Fibrosis

**FIGURE 4:**
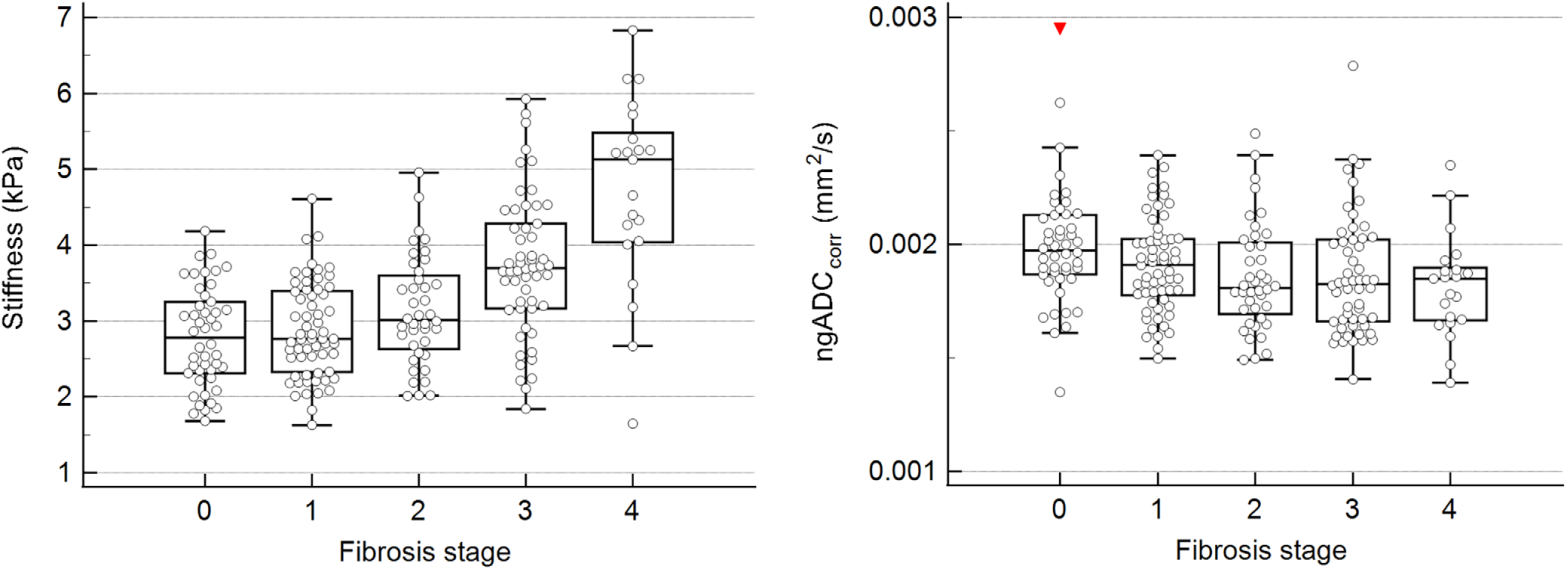
Kruskal–Wallis plots of stiffness versus fibrosis stage on the left (*p* < 0.05) and ngADCcorr versus fibrosis stage on the right (*p* < 0.05). Boxes extend from first to third quartile, with the line indicating the median and whiskers are defined as upper and lower adjacent values.

**FIGURE 5:**
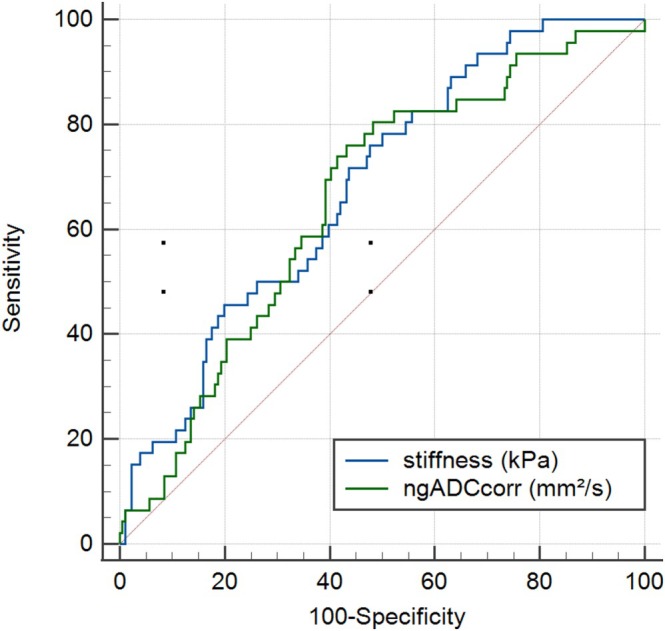
Comparison of ROC curves for stiffness (AUC = 0.68 [0.62–0.74]) and ngADC_corr_ (AUC = 0.66 [0.59–0.71]) in evaluating F0 versus F1234.

The relationships between stiffness and fibrosis and between ngADC_corr_ and fibrosis are shown on the box plots of Figure 4. Stiffness increased significantly across fibrosis stages, while ngADC_corr_ decreased significantly. Post hoc analysis showed that participants without liver fibrosis (F0) had significantly higher ngADC_corr_ than patients with liver fibrosis (F1–F4). ROC curve analysis showed a significant difference (*p* < 0.05) in discriminating F0 versus (vs) F1–F4 with ngADC_corr_ and stiffness (AUC = 0.66 [0.59–0.71] and AUC = 0.68 [0.62–0.74], respectively; Figure 5). For *F* > 1, *F* > 2, *F* > 3, *F* = 4, the AUCs of stiffness and ngADC_corr_ progressively diverged, with stiffness showing higher diagnostic performance (Table 3). Furthermore, the DeLong tests comparing the two variables showed statistical significance for the comparisons between F0F1 versus F2–F4, F0–F2 versus F3F4, and F0–F3 versus F4, indicating that stiffness achieved significantly higher diagnostic performance than ngADC_corr_.

## 4 Discussion

In this prospective study in patients with diabetes and MASLD, we showed that a novel non‐Gaussian diffusion‐based marker, ngADC_corr_, has diagnostic value for detecting liver fibrosis. Notably, ngADC_corr_ was a statistically significant marker of liver fibrosis both at univariate and at multivariate analysis, in contrast to index‐based and uncorrected fit‐based ADCs. This finding shows the benefit of using a fit that includes multiple *b* values, and the importance of appropriately compensating for the presence of steatosis to enhance sensitivity to hepatic fibrosis in MASLD. Although adding b‐values required for the computation of this non‐Gaussian diffusion‐based marker comes at the expense of longer acquisition time relative to the only two *b* values required in the sADC method, it retains the advantage of not requiring an external actuator, as MRE does.

Previously, Kromrey et al. reported good diagnostic performance for sADC, an index‐based marker, for predicting liver stiffness in patients with chronic liver disease [13]. While these results were promising, their generalizability to MASLD remained unknown because of the limited number of patients with MASLD in their study. In a later study performed in a population of MASLD patients, Hanniman et al. reported that sADC did not accurately predict MRE stiffness or histologically determined fibrosis stage because of the confounding effect of steatosis [18]. In liver steatosis, intrahepatocytic fat, besides providing additional diffusion barriers, also directly alters the diffusion‐weighted MRI signal because incomplete fat suppression schemes introduce signal modulations involving the different transverse relaxation rates of water and fat [16, 17]. Therefore, it has been proposed to perform the sADC calculation in two steps, by first calculating the water contribution to the signal and then performing the sADC computation on the corrected water signal. However, application of this fat correction method by Hanniman et al. still did not show a correlation between MRE liver stiffness or histologically determined fibrosis and sADC. Le Bihan proposed a different fat‐correction scheme to correct for the underestimation of sADC in the formulation proposed by Hanniman, but this solution could not be tested because of the absence of a b0 image in Hanniman's dataset [19].

In this study, there was no significant relation in multivariate analysis between fibrosis and sADC, ${\text{sADC}}_{\text{corr}}^{\text{Hanniman}}$ or ${\text{sADC}}_{\text{corr}}^{\text{LeBihan}}$, although the univariate performance of ${\text{sADC}}_{\text{corr}}^{\text{LeBihan}}$ performed slightly better. These results are consistent with findings from Hanniman and colleagues [18]. This negative finding persisted despite having tested both fat correction schemes currently proposed in the literature [18, 19]. The proposed ngADCcorr approach may have proven superior due to its inclusion of information from a greater number of b values and its use of a nonlinear model to better conform to the expected MR signal response to increasing diffusion weightings.

The diagnostic performance of our proposed non‐Gaussian diffusion‐based marker ngADC_corr_ remains limited and is only significant for discriminating F0 from higher fibrosis stages, but does not perform as well for discriminating more advanced stages. Still, the non‐Gaussian diffusion‐based parameter ngADC_corr_ may have some diagnostic utility for fibrosis in a MASLD population. Although MRE estimations of liver stiffness are often considered superior to diffusion parameters for distinguishing fibrosis stages, we observed similar diagnostic performance between ngADC_corr_ and stiffness for diagnosing > F0 [29].

In MASLD, the volume and composition of the extra‐ and intra‐hepatocyte diffusion compartments change during fibrosis progression. Liver steatosis tends to progress in a biphasic manner, increasing in the early stages of MASLD, then decreasing when a larger fraction of the extracellular space is occupied by fibrosis and when liver function is performed by a decreasing number of energy‐starved hepatocytes [30, 31, 32]. Our results are compatible with this type of progression along two phases (Figure S1). In the first phase F0–F2, accumulation of steatosis and fibrosis contributes to a decrease in ADC. In the second phase F3–F4, steatosis decreases, with a partial effect of increasing ADC, but fibrosis continues to accumulate, favoring an opposed effect of decreasing the ADC. This could explain why ADC decreases rapidly in early fibrosis and less later on.

These observations are in line with earlier reports such as that of Annet et al., who showed that liver DWI signals can be significantly affected by both fat and perfusion, especially at low *b*‐values, thereby limiting the reliability of diffusion parameters for fibrosis assessment [33]. These confounding effects may also have contributed to the inconsistent diagnostic performance of DWI in chronic liver disease since earlier reports [34, 35].

Our study had several limitations. Firstly, we estimated the PDFF values by averaging values over different parts of the liver. The PDFF of the region of interest used for diffusion calculations may have been slightly different, introducing some level of variability. While real, PDFF variations across the organ have been evaluated to be small in a study of MASLD patients [36].

Secondly, we used a single diffusion acquisition to obtain short acquisition times. This precluded the application of a quality criterion based on a standard deviation smaller than 15% of the mean signal, as reported by the Le Bihan group [13]. This may partially explain the lower diagnostic performance observed for the sADC‐based indexes in our work relative to the reports of Le Bihan. Future studies should include several diffusion acquisitions to enable the application of a quality criterion or devise quality criteria based on single diffusion acquisitions.

Finally, this was a single‐center study performed on a single scanner from a single vendor and at a single field strength. These conditions, while ensuring technical consistency, limit the generalizability of our findings. Validation across different centers, vendors, and field strengths will be necessary to confirm the robustness and reproducibility of our method.

## 5 Conclusion

The application of the diffusion MRI method we are proposing, involving a non‐Gaussian model, a non‐linear fitting procedure with multiple b values, and fat correction, enabled us to detect liver fibrosis in a MASLD population. The implementation of a correction scheme advantageously reduced the sensitivity of ADC to steatosis. In our study, the method had similar diagnostic performance as MRE for F0 versus F1–F4 staging. If these results are confirmed in other study groups, fat‐corrected non‐Gaussian diffusion might be useful to detect liver fibrosis in MASLD patients.

## Supporting information


**Figure S1:** Kruskall–Wallis plots of PDFF vs. fibrosis stage (*p* = 0.01). Boxes extend from first to third quartile, with the line indicating the median and whiskers are defined as upper and lower adjacent values. This illustrates the biphasic effect of steatosis. In stages F0–F2, fibrosis and steatosis evolve in the same direction, while in stages F3–F4, the evolution of steatosis and fibrosis oppose each other.

**Figure S2:** Comparison of ROC curves for stiffness and ngADC_corr_ in the evaluation of fibrosis severity. From left to right: F01 versus F234, F012 versus F34, and F0123 versus F4.

**Table S1:** Kruskal–Wallis and post hoc pairwise comparisons of stiffness and diffusion parameters across fibrosis stages.

**Table S2:** Kruskal–Wallis and post hoc pairwise comparisons of stiffness and diffusion parameters across steatosis grades.

## Data Availability

The data that support the findings of this study are available from the authors, but restrictions apply to the availability of these data. Data can be made available upon reasonable request after approval by the QuidNASH steering committee.
