# Supplementary figures and images for: Fat‐Corrected Non‐Gaussian Diffusion MRI for Liver Fibrosis Assessment in Metabolic Dysfunction‐Associated Steatotic Liver Disease
Source: J Magn Reson Imaging. 2025 Oct 24;63(2):497–507. doi: 10.1002/jmri.70148 (PMC12811005; doi:10.1002/jmri.70148)

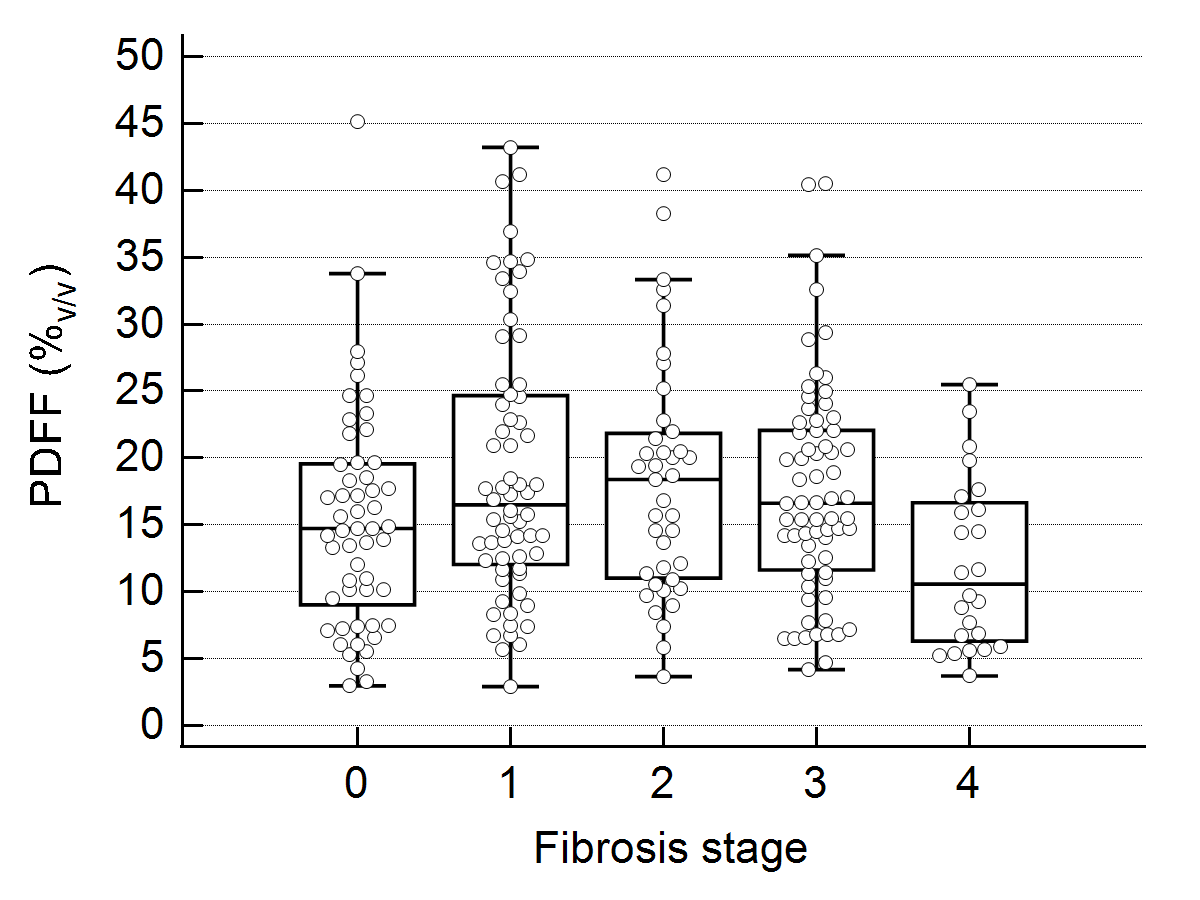

Supplement: Supplementary file 1 — Figure S1: Kruskall–Wallis plots of PDFF vs. fibrosis stage (p = 0.01). Boxes extend from first to third quartile, with the line indicating the median and whiskers are defined as upper and lower adjacent values. This illustrates the biphasic effect of steatosis. In stages F0–F2, fibrosis and steatosis evolve in the same direction, while in stages F3–F4, the evolution of steatosis and fibrosis oppose each other. [file JMRI-63-497-s003.png]

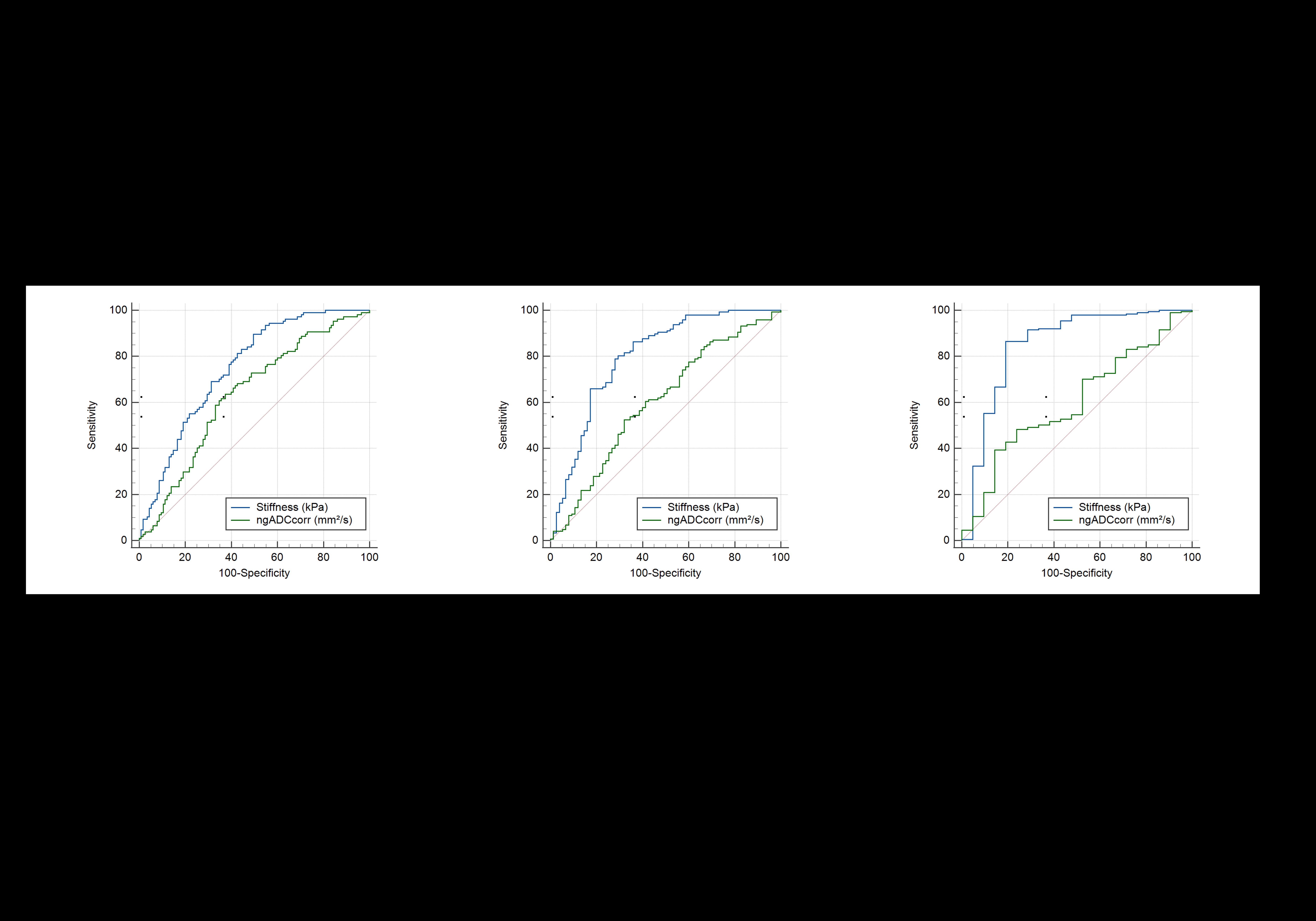

Supplement: Supplementary file 2 — Figure S2: Comparison of ROC curves for stiffness and ngADCcorr in the evaluation of fibrosis severity. From left to right: F01 versus F234, F012 versus F34, and F0123 versus F4. [file JMRI-63-497-s002.png]
