# Supplementary material for: Fat‐Corrected Non‐Gaussian Diffusion MRI for Liver Fibrosis Assessment in Metabolic Dysfunction‐Associated Steatotic Liver Disease
Source: J Magn Reson Imaging. 2025 Oct 24;63(2):497–507. doi: 10.1002/jmri.70148 (PMC12811005; doi:10.1002/jmri.70148)
Supplement: Supplementary file 3 — Table S1: Kruskal–Wallis and post hoc pairwise comparisons of stiffness and diffusion parameters across fibrosis stages. [file JMRI-63-497-s001.docx]

| MRI parameter | Kruskal-Wallis  p-value | F0 vs F1 | F0 vs F2 | F0 vs F3 | F0 vs F4 | F1 vs F2 | F1 vs F3 | F1 vs F4 | F2 vs F3 | F2 vs F4 | F3 vs F4 |
| --- | --- | --- | --- | --- | --- | --- | --- | --- | --- | --- | --- |
| Stiffness | p < 0.05 | 1.00 | 0.31 | p<0.05 | p<0.05 | 0.74 | p<0.05 | p<0.05 | p<0.05 | p<0.05 | 0.10 |
| sADC | p = 0.08 | _ | _ | _ | _ | _ | _ | _ | _ | _ | _ |
| $\mathrm{sADC}_{\mathrm{corr}}^{\mathrm{Hanniman}}$ | p = 0.08 | _ | _ | _ | _ | _ | _ | _ | _ | _ | _ |
| $\mathrm{sADC}_{\mathrm{corr}}^{Le Bihan}$ | p = 0.06 | _ | _ | _ | _ | _ | _ | _ | _ | _ | _ |
| ngADC | p = 0.11 | _ | _ | _ | _ | _ | _ | _ | _ | _ | _ |
| $\mathrm{ngADC}_{\mathrm{corr}}$ | p < 0.05 | 0.84 | p<0.05 | p<0.05 | p<0.05 | 1.00 | 1.00 | 0.99 | 1.00 | 1.00 | 1.00 |
| ADC mono-exponential | p=0.20 | _ | _ | _ | _ | _ | _ | _ | _ | _ | _ |

Table S1 : Kruskal–Wallis and post hoc pairwise comparisons of stiffness and diffusion parameters across fibrosis stages
