# Supplementary material for: Fat‐Corrected Non‐Gaussian Diffusion MRI for Liver Fibrosis Assessment in Metabolic Dysfunction‐Associated Steatotic Liver Disease
Source: J Magn Reson Imaging. 2025 Oct 24;63(2):497–507. doi: 10.1002/jmri.70148 (PMC12811005; doi:10.1002/jmri.70148)
Supplement: Supplementary file 4 — Table S2: Kruskal–Wallis and post hoc pairwise comparisons of stiffness and diffusion parameters across steatosis grades. [file JMRI-63-497-s004.docx]

Table S2 : Kruskal–Wallis and post hoc pairwise comparisons of stiffness and diffusion parameters across steatosis grades

| MRI parameter | Kruskal-Wallis  p-value | S0 vs S1 | S0 vs S2 | S0 vs S3 | S1 vs S2 | S1 vs S3 | S2 vs S3 |
| --- | --- | --- | --- | --- | --- | --- | --- |
| Stiffness | p = 0.84 | _ | _ | _ | _ | _ | _ |
| sADC | p<0.05 | 0.13 | p<0.05 | p<0.05 | p<0.05 | p<0.05 | p<0.05 |
| $\mathrm{sADC}_{\mathrm{corr}}^{\mathrm{Hanniman}}$ | p = 0.13 | _ | _ | _ | _ | _ | _ |
| $\mathrm{sADC}_{\mathrm{corr}}^{Le Bihan}$ | p = 0.13 | _ | _ | _ | _ | _ | _ |
| ngADC | p<0.05 | 0.23 | p<0.05 | p<0.05 | ns | p<0.05 | p<0.05 |
| $\mathrm{ngADC}_{\mathrm{corr}}$ | p = 0.98 | _ | _ | _ | _ | _ | _ |
| ADC mono-exponential | p<0.05 | 0.62 | p<0.05 | p<0.05 | p<0.05 | p<0.05 | p<0.05 |
